# A NIR-Based Study of Desorption Kinetics during Continuous Spin Freeze-Drying

**DOI:** 10.3390/pharmaceutics13122168

**Published:** 2021-12-16

**Authors:** Laurens Leys, Gust Nuytten, Joris Lammens, Pieter-Jan Van Bockstal, Jos Corver, Chris Vervaet, Thomas De Beer

**Affiliations:** 1 Laboratory of Pharmaceutical Process Analytical Technology, Department of Pharmaceutical Analysis, Ghent University, 9000 Ghent, Belgium; laurens.leys@ugent.be (L.L.); Gust.Nuytten@ugent.be (G.N.); 2 Laboratory of Pharmaceutical Technology, Department of Pharmaceutics, Ghent University, 9000 Ghent, Belgium; Joris.Lammens@ugent.be (J.L.); Chris.Vervaet@ugent.be (C.V.); 3 RheaVita, Technologiepark-Zwijnaarde 3, Postbus 17, Zwijnaarde, 9052 Ghent, Belgium; pieterjan.vanbockstal@ugent.be (P.-J.V.B.); j.corver@rheavita.com (J.C.)

**Keywords:** freeze-drying, continuous manufacturing, desorption, secondary drying, continuous freeze-drying, near-infrared

## Abstract

The pharmaceutical industry is progressing toward the development of more continuous manufacturing techniques. At the same time, the industry is striving toward more process understanding and improved process control, which requires the implementation of process analytical technology tools (PAT). For the purpose of drying biopharmaceuticals, a continuous spin freeze-drying technology for unit doses was developed, which is based on creating thin layers of product by spinning the solution during the freezing step. Drying is performed under vacuum using infrared heaters to provide energy for the sublimation process. This approach reduces drying times by more than 90% compared to conventional batch freeze-drying. In this work, a new methodology is presented using near-infrared (NIR) spectroscopy to study the desorption kinetics during the secondary drying step of the continuous spin freeze-drying process. An inline PLS-based NIR calibration model to predict the residual moisture content of a standard formulation (i.e., 10% sucrose) was constructed and validated. This model was then used to evaluate the effect of different process parameters on the desorption rate. Product temperature, which was controlled by a PID feedback mechanism of the IR heaters, had the highest positive impact on the drying rate during secondary drying. Using a higher cooling rate during spin freezing was found to significantly increase the desorption rate as well. A higher filling volume had a smaller negative effect on the drying rate while the chamber pressure during drying was found to have no significant effect in the range between 10 and 30 Pa.

## 1. Introduction

The interest toward continuous manufacturing of biopharmaceuticals has gradually increased during the past two decades. The advantages of continuous manufacturing over batch manufacturing are numerous, including faster processing times, improved product quality, and various new possibilities for enhanced quality control [1,2]. In addition, since the drying of these products often requires a gentle and low temperature drying process to improve the long-term stability of these products, there is a special interest toward the realization of an end-to-end continuous freeze-drying manufacturing method [3,4,5,6]. For this purpose, a continuous spin freeze-drying technology was developed for unit doses. Using this technology, the vials with liquid drug solution are rapidly spun along their axis during the freezing step using a cold inert gas to freeze the product. By doing so, a hollow cylindrical frozen product is obtained on the walls of the vial. The spin-frozen vials are subsequently transferred to a continuous vacuum drying chamber using a load-lock system to maintain pressure control between the two process stages. In this drying chamber, vials are continuously moved and rotated in front of several IR-heaters placed in series to speed up the sublimation process. Due to the spin-freezing step, the layer thickness of the product is drastically decreased and a larger surface area is obtained allowing fast sublimation of the ice crystals reducing drying times by more than 90% compared to a batch freeze-drying process [7,8,9]. These drying times can be improved even more using mechanistic models to calculate the optimal process settings during the drying process [9,10].

Over the last several years, the advantages of the continuous spin freeze-drying technology were discussed in several research papers, covering mainly topics concerning the primary drying step of the process [8,9,10,11,12,13,14]. However, after primary drying, a considerable amount of moisture remains present inside the freeze-dried product, which can be as high as 8%–15% depending on the product. This moisture is considered to be dissolved in or bound to the dried product and can be removed by a secondary drying process. To remove this residual water, a higher product temperature is needed while vacuum conditions are maintained. Since the stability of the final product is highly dependent on the residual moisture content and secondary drying times can take up a significant portion of the total processing time, this process step needs to be thoroughly understood. For batch freeze-drying, studies have been conducted in the past to examine the effect of process parameters on the secondary drying process [15,16]. These studies found that not only the shelf temperature during secondary drying but also predrying steps can have a significant impact on the desorption rate. It was observed that using controlled nucleation during the freezing phase or introducing an annealing step generally leads to a lower specific surface area of the product, which has a negative impact on the desorption rate [16,17,18]. The effect of cake thickness and chamber pressure was negligible [15]. This led to the conclusion that the rate-determining step for the desorption rate in batch freeze-drying process is most likely controlled by one of the following steps: (1) diffusion of water from the inside of the solid matrix to the solid–vapor interface (pores) or (2) evaporation of the water at the solid–vapor interface. The transport of the water vapor through the pores of the dried cake was deemed not rate-limiting since this mass transfer mechanism would be influenced by the cake thickness or chamber pressure as is observed during primary drying. For spin freeze-drying, however, no such studies have been conducted yet. Since both the freezing phase and drying phase of this process are remarkably different from a batch freeze-drying process, changes in the desorption mechanism might occur due to alterations in the pore structure or heat transfer mechanism (IR).

To study the desorption rate during the continuous spin freeze-drying process, a new methodology that is compatible with the technology is needed. In the past, near-infrared (NIR) spectroscopy has been used to monitor the drying process during spin freeze-drying using principal component analysis (PCA) to analyze the spectra. From these data, the endpoint of the primary and secondary drying process could be derived. Nonetheless, no quantitative results were obtained from the data using this approach, meaning that the residual moisture content during the desorption process was not obtained [11,12,19]. Brouckaert et al., on the other hand, established a quantitative multivariate curve resolution model based on near-infrared chemical imaging (NIR-CI) to determine the water content and the solid-state properties of the final spin freeze-dried product. It was proved that NIR-CI can be used as a powerful process analytical tool (PAT) for quality control. However, an offline setup was used in this work due to some practical limitations for inline implementation of the NIR-CI camera [20]. Therefore, in this work, an inline NIR method was developed to derive the residual moisture value inline using partial least square analysis (PLS). The evolution of the residual moisture decrease during secondary drying could be quantitatively monitored allowing to study the desorption kinetics during continuous freeze-drying. Using this approach, influential process parameters could be identified and quantified using a design of experiments (DOE) approach.

## 2. Materials and Methods

### 2.1. Development of the Inline NIR-Based Moisture Quantification Model

#### 2.1.1. Sample Preparation and Spin Freeze-Drying Setup

A solution of 10% (*w*/*v*) sucrose (Merck, Overijse, Belgium) in water was chosen as model formulation for the development of the NIR-based moisture quantification model. 10R vials (Schott, Müllheim, Germany), filled with 4 mL of this formulation, were spun using a WB6000-D high-speed overhead stirrer (Wiggens, Beijing, China), which rotated at 4000 rpm. Meanwhile, the formulation was frozen using a cold airstream, which was cooled by a liquid nitrogen heat exchanger. A flow rate of 25 L/min was used. The gas temperature was approximately −60 °C. Next, all vials were transferred to cylindrical aluminum holders, which were precooled by placing them on the shelves (−40 °C) of an Amsco FINNAQUA GT4 freeze-dryer (GEA, Köln, Germany). Multiple freeze-drying runs were performed with varying secondary drying conditions to obtain samples with different moisture contents. Primary drying was always performed using a shelf temperature of −30 °C and a chamber pressure of 10 Pa. The endpoint of the primary drying step was detected by comparative pressure measurement after which the secondary drying step was started. The following secondary drying temperatures were used for the different runs: −30, −25, 15, 0, 20, and 40 °C. A secondary drying time of 8 h was used and the chamber pressure was fixed at 10 Pa. After secondary drying, samples were stoppered under vacuum and the chamber was backfilled with nitrogen gas. All samples were placed on dry ice after process completion to preserve the samples for the next step (Section 2.1.2). This resulted in a total of 54 predried samples with moisture contents varying between 0% to 8%.

#### 2.1.2. Near-Infrared Spectroscopy

**Figure 1:**
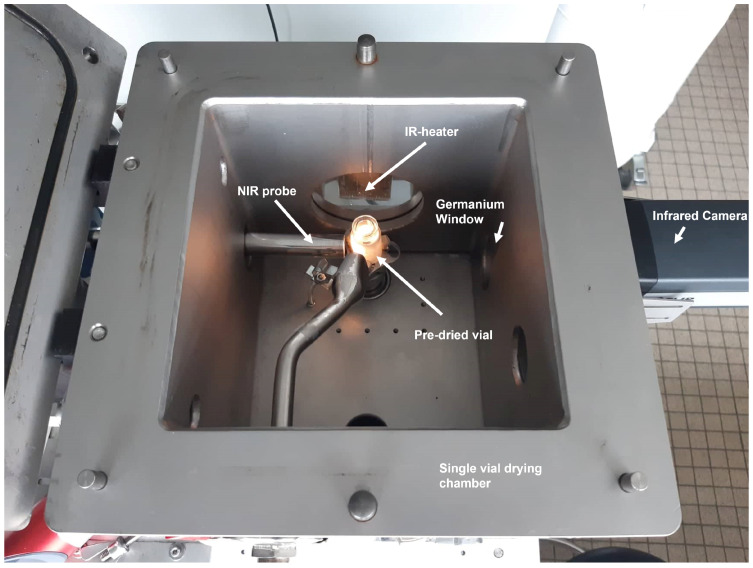
Single vial drying chamber and experimental setup for NIR measurements with product temperature control.

The 54 predried samples were divided in two groups; one was used as a PLS calibration set while the other was used as a validation set. The calibration set consisted of 46 samples and the validation set of 8 samples. Samples were placed inside the gripper of the single vial continuous freeze-drying prototype (RheaVita, Zwijnaarde, Belgium) in front of a SentroProbe DLR RS NIR FO (Sentronic, Dresden, Germany) as depicted in Figure 1. This probe was connected to a SentroPAT FO diode array system using a fiber optic cable. It should be noted that the single vial system is an R&D simulation system for one vial, which is able to spin freeze and dry in the same chamber. Per sample, 10 spectra were collected in the 1100–2200 nm region with an averaging number of 125 and an integration time of 10 ms. This was done from the side of the glass vial under vacuum while the vial was rotating at a speed of 5 rpm in order to take into account the spatial differences in moisture content. After spectrum collection, the vial was removed from the freeze-drying unit and a Karl Fischer assay (KF) was performed on the sample to obtain the residual moisture content.

#### 2.1.3. PLS Calibration

The collected NIR spectra from the 46 calibration samples were linked to their residual moisture content (determined via KF) using the multivariate data analysis software SIMCA 16.0.2 (Umetrics, Umeå, Sweden). All spectra were smoothed by applying a Savitsky–Golay (SG) filter, which fitted a quadratic polynomial function over a moving data subset of 15 data points. Standard normal variate (SNV) preprocessing was performed to eliminate baseline offset variations between the samples caused by small differences in probe to product distance. Several calibration models were constructed varying in wavelength range. For the first model, the full collected spectral range was used (1100–2200 nm), while for the other models, two smaller wavelength regions were selected ranging from 1390–2200 nm and 1865–2200 nm. The optimal number of latent variables (LV) for every model was selected based on the root mean square error of cross-validation (RMSEcv). Latent variables were added until the RMSEcv did not significantly decrease anymore. The accuracy of the resulting PLS models was evaluated and compared by calculating the RMSEcv for the calibration set and the root mean square error of prediction (RMSEP) for the validation set. The most accurate model was used for further experiments.

### 2.2. Determination of the Influential Parameters on Desorption Kinetics

#### 2.2.1. Sample Preparation

A total of 11 samples were prepared as explained in Section 2.1.1. Filling volume of the vial (10R) was varied between 2 and 4 mL. The cooling rate, which is defined as the rate at which the vial temperature decreases during the initial cooling phase (i.e., before nucleation), was regulated using proportional–integral–derivative (PID) control of a Bronkhorst^®^ F-203AV digital mass flow controller (Flowcor, Belgium) on the cold gas stream. The temperature of the vial was monitored during this initial cooling phase using a FLIR A655sc IR camera (Thermal Focus, Ravels, Belgium) to provide the input for the PID control algorithm, which was programmed in a custom-made Labview^®^ 2018 program. Upon nucleation, the freezing phase starts, which is defined as the phase at which ice crystals are formed. This phase was controlled using the same heat transfer rate over the vial as in the initial cooling phase ($\dot{Q_{c}}$). If a constant cooling rate is used during the initial cooling phase, $\dot{Q_{c}}$ is a constant as can be derived from following formula:

(1) $$\dot{Q_{c}}=C_{r}C_{tot}$$

where $C_{r}$ is the cooling rate during the initial cooling phase (K/min) and $C_{tot}$ (J/K) is the combined total heat capacity for the vial and the liquid. Heat transfer rate over the vial during the freezing phase ($\dot{Q_{fr}}$) was calculated using Equation (2):

(2) $$\dot{Q_{fr}}=2\pi r_{v}Lh\left(T_{v}-T_{gas}\right)$$

where $r_{v}$ is the radius of the vial (m), L is the height of the vial (m), and $T_{v}$ and $T_{gas}$ (K) are the temperature of the vial and the gas, respectively. The heat transfer coefficient *h* (W/m^2^K) is dependent on the flow rate of the cold gas. For this reason, *h* was calibrated beforehand in function of the gas flow rate using linear regression. A type-K thermocouple (Conrad Electronic, Hirschau, Germany) was used to measure the gas temperature at the outlet of the gas diffuser during the freezing phase while the FLIR was used to measure the vial temperature. By monitoring these two temperatures, the gas flow could continuously be adjusted by the Labview^®^ program in order to change *h* so that $\dot{Q_{fr}}$ equals $\dot{Q_{c}}$ at all time during the freezing phase. Using this approach, the initial cooling phase is linked to the freezing phase ensuring that low cooling rates will be accompanied by slow freezing rates and vice versa. Cooling rates were varied between 9 and 50 K/min according to the experimental design (see Section 2.2.4). After freezing, all vials were predried in the batch freeze dryer using a shelf temperature of −30 °C and a chamber pressure of 10 Pa. The process was interrupted when primary drying was completed to achieve a high residual moisture content (±8%) for every vial. The endpoint of primary drying was detected using comparative pressure measurement. Samples were immediately placed on dry ice after the predrying step to inhibit further desorption.

#### 2.2.2. NIR Measurement

Secondary drying was performed for every sample in the single vial continuous freeze-drying prototype. Each sample was placed inside the grippers in front of the NIR probe. After closing the lid, the chamber pressure was immediately lowered to the required level. A desired product temperature was set and controlled by a PID feedback system of the power supply of the IR heater. The PID feedback system was programmed in Labview^®^ 2018, which used the product temperature measurement, provided by a FLIR A655sc IR camera, to provide the input for the PID algorithm. Set values for product temperature and pressure were varied between 30 to 40 °C and 10 to 30 Pa, respectively, according to the experimental design. During the desorption process, one spectrum was collected for every vial every minute for a total drying time of 2 h. After 2 h, all spectra were collected and analyzed using SIMCA 16.0.2. The residual moisture content at every time point was predicted using the most accurate PLS model. In addition, the moisture content at the end of every drying experiment was measured by Karl Fischer and was compared with the prediction of the PLS model to verify that the drying process did not influence the accuracy of the model predictions.

#### 2.2.3. Mathematical Description of the Desorption Process

The desorption process is often described by the following empirical equation [21,22,23]:

(3) $$\frac{dC}{dt}=-k_{d}\left(C-C_{eq}\right)$$

where *C* (%) is the residual moisture content at time *t* (s) and $k_{d}$ (1/s) is the kinetic desorption constant. $C_{eq}$ is the equilibrium moisture content, which can be considered zero when relatively high product temperatures are used inside a vacuum chamber, as is the case in this work [24]. The solution of this equation has the following form and is a small adaptation of Lewis (Newton) drying model for thin layers, which is commonly used to describe convective drying processes in the food industry [25,26].

(4) $$C\left(t\right)=C_{0}e^{-k_{d}t}$$

where $C_{0}$ is the initial moisture content at the beginning of the desorption process. However, it was found by Sahni and Pikal that Equation (5) provides a more accurate description of the desorption process [22].

(5) $$\frac{dC}{d\sqrt{t}}=-k_{d}C$$

The solution of Equation (5) is a specific version of the Page model, which is widely and successfully used to describe the drying of thin layers of fruits [27,28]:

(6) $$C\left(t\right)=C_{0}e^{-k_{d}\sqrt{t}}$$

MATLAB R2018b (Mathworks, Natick, MA, USA) was used to fit the data to the empirical model and obtain $k_{d}$.

#### 2.2.4. Experimental Design

In order to identify the significant influential parameters on desorption kinetics during continuous spin freeze-drying, a design of experiments approach was used. The factors that were examined in this study were filling volume, cooling rate, and chamber pressure and product temperature during drying. A fractional factorial screening design was selected, which resulted in 11 experiments including 3 center points. An overview of the conducted experiments and the factor set points is given in Table 1. Two responses were selected to evaluate the desorption kinetics of the different runs. The first response was the desorption parameter $k_{d}$, which was determined as described in Section 2.2.3. The second response was the predicted (NIR) moisture content of the product after 2 h of secondary drying $C_{end}$. To analyze the results, Modde Pro 12.1 (Umetrics, Umeå, Sweden) was used. A skewness test was performed on the responses, which were log-transformed if needed. A multiple linear regression (MLR) model was fitted through the results. Regression coefficients for interactions between factors were only added if the coefficient significantly increased the fraction of explained responses ($R^{2}$) and predictability ($Q^{2}$) of the model. The 95% confidence interval was calculated for every regression coefficient and was considered significant if the interval did not contain zero.

## 3. Results and Discussion

### 3.1. Development and Validation of the PLS Calibration Model

**Figure 2:**
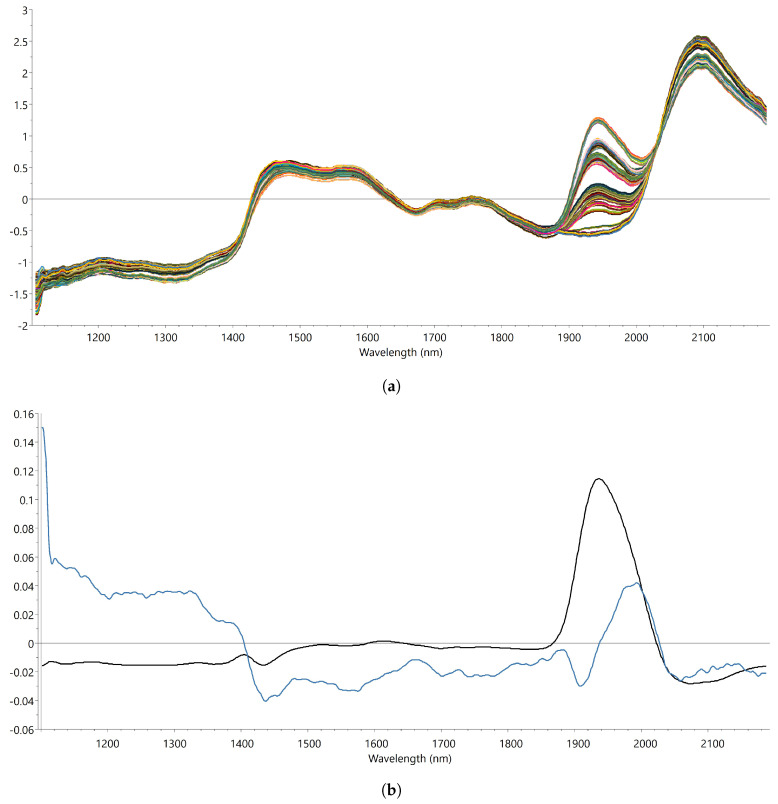
(**a**) SNV-SG filtered spectra (calibration set); (**b**) loadings of PLS calibration model (range 1100–2200 nm); black line (LV1), blue line (LV2).

To obtain the most accurate model for further experiments, 3 PLS calibration models were constructed varying in wavelength range. Figure 2a shows the spectral data of the full wavelength region after preprocessing (SNV-SG) for all samples of the calibration set. It is clear that most of the variation in spectral variability can be found around the region of 1940 nm. This band can be attributed to the combination of O-H stretching and O-H bending of the water molecules in the sample. The first latent variable (LV1) of the model for which the full spectrum was used (1100–2200 nm) already explained 97.4% of the total variance in residual moisture content between the samples. In addition, since the weights of the loadings of LV1 (Figure 2b) are highest in the water band region (1860–2040 nm), the model can be considered highly specific for the residual water content of the sample. To improve the accuracy of this first model, smaller wavelength regions were selected focusing more on the O-H stretching and O-H bending band of the spectrum. As can be seen from Table 2, the RMSEcv and RMSEP of the models with a smaller spectral range is smaller than for the model where the full wavelength range was used, indicating a higher accuracy for the smaller wavelength region models. Furthermore, the RMSEcv and RMSEP did not significantly decrease any further after addition of a second LV. Therefore, the number of LVs for all models was fixed at 2 to avoid overfitting of the data. It seems likely, since PLS is a linear regression technique, that adding a second component corrects for the nonlinear behavior of the absorbance in the 1940 nm region versus the water content. This is indicated by the importance of the weights of LV2 in the region of 1800–2050 nm, which is also located in the region of the aforementioned combination band of water.

**Figure 3:**
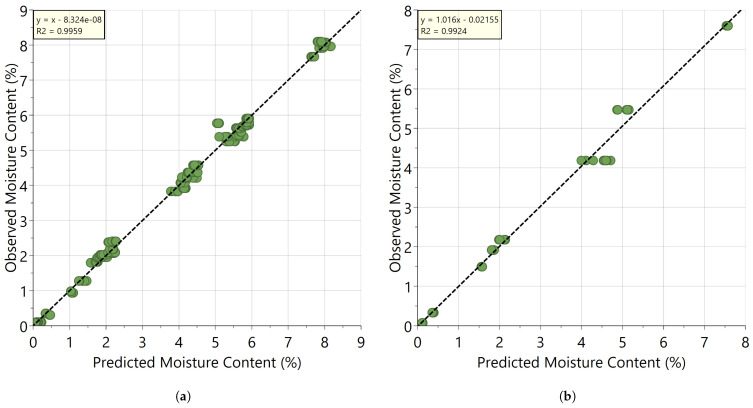
Observed residual moisture content versus predicted plot using SNV-SG corrected data in the range of 1865–2200 nm: (**a**) calibration set; (**b**) validation set.

The model with the lowest RMSEcv and RMSEP was selected for further use, which is the model using the wavelength range of 1865 to 2200 nm. As can be seen from Figure 3a,b, a high correlation (>0.99) can be observed between the observed residual moisture content (i.e., with KF) and the predicted moisture content by the NIR method for both the calibration and validation samples. The slope of these plots is 1.0 and the intercept close to zero indicating that there is no relative or absolute bias of the model. The calculated RMSEcv and RMSEP for the model, respectively 0.161% and 0.216%, indicate that the accuracy of the PLS calibration model is sufficient for inline moisture determination.

### 3.2. Fitting of the Drying Curves and Validation of the Inline Predictions

**Figure 4:**
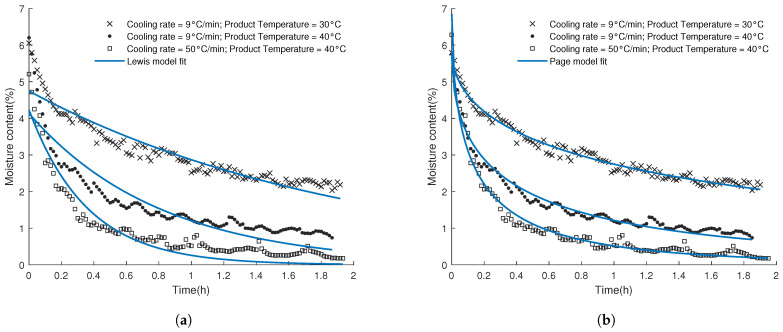
NIR-based inline moisture content prediction in function of time for run 5, 7, and 8; Fitting of the data was done using either the Lewis (**a**) or Page (**b**) drying model.

In order to not only compare the effect of different process settings on the final moisture content ($C_{end}$), a descriptive parameter for the rate of desorption is needed. In literature, the kinetic desorption rate constant, $k_{d}$, is often used by fitting the rate of desorption over time with Equation (3). In this work, however, a fast initial decline in moisture content was observed followed by a sudden decrease in the drying rate, which results in a poor fit of the Lewis model (Equation (4)). As can be seen in Figure 4, the Lewis model tends to underestimate the drying rate in the early phase and overestimate the drying rate at the end of the drying process. Page’s model (Equation (6)) adjusts the model of Lewis by adding a power term (*n*) to the time term in the Lewis model. Since log(C) seems to be linear in function of the square root of time (*n* = 0.5) for all the performed experiments, Page’s model was found to provide a better description of the desorption process for spin-frozen products. Therefore, Page’s model was used to estimate $k_{d}$. It is important to note, however, that the value of $k_{d}$ depends on the initial starting moisture content ($C_{0})$ and declines in function of $C_{0}$. Therefore, fitting was always started from the point where the desorption curve reached a moisture content of 6%. At this point, the product temperature of all samples had reached its set value by the PID control, thereby eliminating the influence on temperature on $k_{d}$ during the heat-up phase. Finally, to validate the accuracy of the inline predictions during the secondary drying process, the real residual moisture content at the end of the process of every sample (i.e., determined with KF) was compared with the predictions of the PLS model. As can be seen from Table 1, the value of the KF measurement was always within the 95% prediction interval (±2*RMSEcv) of the used PLS calibration model. It was therefore concluded that drying in the single vial prototype did not cause any physical or chemical changes in the sample which would lead to a change in its spectrum.

### 3.3. Influence of Process Settings on the Desorption Process

**Figure 5:**
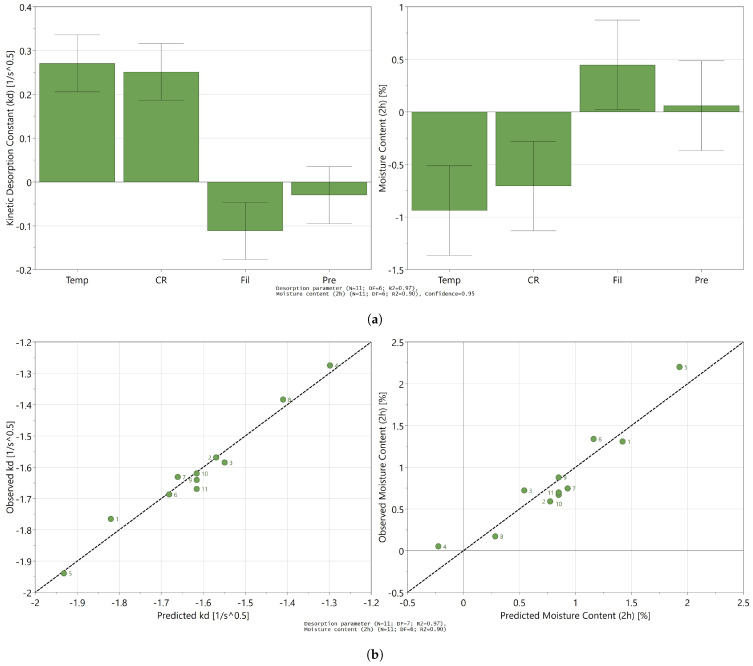
MLR model output. (**a**) Effect plot for of the unrefined MLR models; (**b**) observed vs. predicted responses ($k_{d}$ and $C_{end}$) of the refined MLR models of all performed experimental runs; values of $k_{d}$ were log-transformed.

To determine the influence of the different process settings upon the desorption rate and the final moisture content, a DOE approach was used. Results for the DOE responses, $k_{d}$ and $C_{end}$, are listed in Table 1. The variability of the results for the replicates (center points) was low compared to the overall variability for both responses which is a necessity for constructing a useful MLR model. For the desorption rate parameter $k_{d}$, the skewness test indicated a non-normal distribution of the responses. Therefore, log-transformation was performed on the $k_{d}$ response. Only product temperature, cooling rate, and filling volume had a significant impact on the desorption rate of the product ($k_{d}$) as can be seen from Figure 5a. The pressure did not have a significant impact on the desorption rate when varied in the range of 10 to 30 Pa and was therefore excluded from the model. This resulted in a final MLR model for $k_{d}$ with a $R^{2}$ 0.97 and a $Q^{2}$ of 0.92. A second model was made for the residual moisture content after 2 h of secondary drying to assess the significance of differences in drying rates on the final moisture content. No transformation was needed for this response. The model contained the same significant influential process parameters as for the drying rate as shown in Figure 5a. However, adding an interaction coefficient for temperature and filling volume increased the $R^{2}$ and $Q^{2}$ of the model from 0.89 and 0.60 to 0.97 and 0.82, respectively, and was therefore included. In the end, two highly predictive models could be obtained as can be observed from Figure 5b, which show the observed vs. predicted plots for both responses.

As expected, product temperature had the largest impact on both the desorption rate and the residual moisture content after process completion. This can easily be explained by the fact that if more energy is provided by IR radiation, a higher product temperatures will be obtained, which increases the available energy for desorption. Since the skewness test indicated that log-transformation was needed for the $k_{d}$ response to construct a valid MLR model, this parameter is expected to follow a nonlinear relationship with at least one of the influential factors. It is likely that this is the case for product temperature since the relationship with $k_{d}$ is commonly modeled using an Arrhenius-type relationship for batch freeze-drying processes [21]:

(7) $$k_{d}=k_{d,0}e^{-E_{a,d}/RT}$$

where $E_{a,d}$ is the activation energy for the desorption process (J mol^−1^), *R* is the ideal gas constant (J K^−1^ mol^−1^), and *T* the product temperature (K). The pre-exponential factor, $k_{d,0}$ (1/s), can be regarded as the frequency at which water molecules become available to evaporate from the solid matrix at a standard residual moisture concentration of 1% in the case of surface evaporation rate-limiting conditions. The exponential term $e^{-E_{a,d}/RT}$ is the fraction of water molecules with enough energy to overcome the activation barrier required for evaporation. The cooling rate was also expected to have a significant impact on the desorption rate. The results show that this is indeed the case as the desorption rate is faster for samples that were cooled with a higher cooling rate [18]. For batch freeze-drying processes, similar behavior is commonly observed. Higher cooling rates are typically associated with the formation of smaller ice crystals. This leads to a larger specific surface area of the product, which is beneficial for the desorption process. Larger crystals are typically formed for samples where a lower cooling rate is used or an annealing step is performed, leading to a smaller specific surface area of the product and a slower desorption rate [18]. The filling volume had a small negative impact on the drying rate, which resulted in a slightly higher moisture content at the end of the drying process for the higher filling volumes. Changing the filling volume from 2 to 4 mL increased the residual moisture content with 0.44% using a 2 h secondary drying process and a product temperature of 30 °C. However, using a higher product temperature during drying decreased the significance of this effect to almost zero, explaining the interaction term in the model. This means that even though the desorption rate is slightly slower for samples with a higher filling volume, the final moisture content is not significantly different from samples with a lower filling volume due to the fact that both drying curves reach a comparable plateau value after 2 h of secondary drying at higher product temperatures. From all the studied factors, chamber pressure was the single parameter that neither impacted the drying rate nor the residual moisture content at the end of the secondary drying process. This can be explained by the fact that rate of desorption is not influenced by a pressure gradient in the dried cake, as it observed during primary drying. In other words, vapor transport through the pores of the dried cake is not the rate-limiting step of the desorption mechanism. This has also previously been confirmed for batch freeze-drying processes [15]. Therefore, since the desorption rate seems to depend on the specific surface area of the product, the desorption rate is more likely controlled by the solid matrix. This can either be the diffusion of water inside the solid matrix to the surface of the water–vapor interface in the pores of the dried layer or evaporation from the interface. This means that for both continuous spin freeze-drying and batch freeze-drying processes, the desorption rate can only be enhanced by increasing the product temperature and not by lowering the pressure, which is a common misconception in industry for batch freeze-drying processes.

### 3.4. Toward a Mechanistic Understanding of Desorption

In the pharmaceutical industry, there is a general trend to optimize process settings for all phases of the freeze-drying process by a trial-and-error approach. Since this is a time-consuming, labor-intensive, and costly process, making use of mechanistic models is becoming more and more desired. Especially for the primary drying phase, these models have proved their worth as they accurately describe the process and allow for the construction of a reliable design space for process settings. Secondary drying, on the other hand, is less understood. For batch freeze-drying, the rate-determining step for the secondary drying process is assumed to be the diffusion of water inside the solid material or evaporation from the solid at the pore surface as mentioned before [15]. The conducted experiments show that spin freezing most likely does not change this mechanism. Therefore, a similar desorption model can potentially be derived from batch freeze-drying and applied to continuous spin freeze-drying to combine both heat and mass transfer through the dried layer. Lumped models, as described by Pisano et al., describe the rate of desorption by a single kinetic desorption parameter ($k_{d}$) using Equation (3) (Lewis). The Arrhenius relationship is used to model the dependence of $k_{d}$ on product temperature [21]. However, this approach is most likely not applicable for the continuous spin freeze-drying technology, as this equation does not provide an adequate description of the desorption process as described in Section 3.2. Interestingly, using the Lewis model for modeling the desorption process has been questioned as well for batch freeze-drying processes since it does not accurately describe the desorption process in some cases [22,24]. This already provides some insight in the mechanism of desorption for the spin freeze-drying process. The reason for this is that the Lewis model is a simplification of the solution of Fick’s second law of diffusion in an semi-infinite slab as described by Crank [29]. This means that describing the desorption process by a simple one-dimensional diffusion model where water diffuses from the inside of the solid matrix to surface at the top of the dried layer would not result in accurate predictions of the desorption rate during spin freeze-drying. The initial diffusion model of Pikal et al. (1990) for batch freeze-drying, which was represented by a system of connected particles of different sizes, cannot be used for the same reason as it was based on Crank’s solution for Fickean drying behavior [15]. Models like the one from Millman et al. take into account that secondary drying starts during the primary drying phase from the moment a dry layer is created on top of the sublimating ice [23]. However, they are still based on the assumption of a single desorption kinetic approach. Additionally, the link with freezing rate or nucleation temperature is often lacking. Trelea et al., on the other hand, used a two-compartment model where water can be present in one of two distinctive phases during the secondary drying phase, a monolayer or the multilayer. Each of these layers were assigned a characteristic desorption time, which significantly improved the description of the drying process [24]. These last findings are in close agreement with was presented above, meaning that the desorption process cannot adequately be described by a single desorption kinetic representation. Therefore, the multilayer model of Trelea et al. seems to be a suitable candidate for modeling the desorption process during the secondary drying phase of the continuous freeze-drying technology. Another potential hypothesis that could provide a mechanistic explanation for the non-Fickean drying behavior could be that the rate-limiting step changes during the secondary drying process. It seems plausible that the product at the start of the secondary drying process contains a large amount of adsorbed water at the surface of the pores (water–vapor boundary) due to the large specific surface area of the freeze-dried product, which is immediately available for evaporation. This water can be evaporated quickly when enough energy is provided by the IR-heaters to heat up the product after the sublimation phase (i.e., primary drying) is completed. Due to the fast removal of this surface water, a large concentration gradient is created from the inside of the solid matrix to the solid–vapor boundary and diffusion of water along this gradient becomes the rate-limiting step in a second phase. A last possible hypothesis is that the diffusion of water in the solid matrix is the rate-limiting step and the diffusion coefficient depends on the residual moisture content of solid. This effect could result in a fast decrease of the desorption rate resulting in a plateau effect as is observed in these experiments. Clearly, providing an explanation for the behavior of the drying process is not an easy task as the experimental verification of these hypotheses is difficult. However, using inline NIR spectroscopy might prove to be a valuable tool for the clarification and calibration of future descriptive models.

### 3.5. Applicability of the Presented Approach

**Figure 6:**
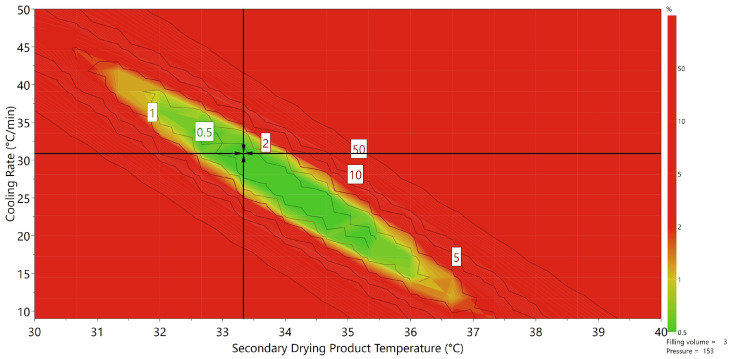
Design space for the significant process settings cooling rate and product temperature. All combinations of process settings in the green area result in a final moisture content between 0.8% and 1.2% with an acceptance value of 1% (filling volume = 3 mL, chamber pressure 15.3 Pa). Red regions indicate combination of process settings, which will lead to acceptance values higher than 1%; the most robust setpoint is indicated by the black arrow cross.

The presented approach, which makes use of inline NIR measurements, proved a reliable and accurate method to study desorption kinetics during freeze drying. The strength of this methodology lies in the amount of data this approach can acquire without affecting the drying process. The conventional approach, which is often used for batch freeze-drying processes, makes use of timely collections of samples out of the chamber or microbalance techniques. The first approach is hampered by the fact that no desorption profile of a single sample can be acquired. The second approach interferes with the drying process since the vial is lifted off the shelf for mass measurement, therefore interrupting the heat transfer from the shelf to the vial. The NIR measurement, used in this work, has the advantage that residual moisture values can be acquired from a single sample at a desired time interval while heat transfer to the vial remains unaffected. NIR has been successfully used in the past to study the different phases of batch freeze-drying processes or as a quality assurance tool to measure the moisture content after secondary drying. However, to our knowledge, NIR has never been used to study the effect of process settings on the drying rate. This approach is especially advantageous for the continuous spin freeze-drying technique used in this work. Since the product is located on the side of the vial and the vial is rotating in front of the probe, not only can the drying rate be studied, but the homogeneity of the moisture content in the product can also be studied over time as well. In addition, the conventional techniques, mentioned above for batch freeze-drying, are not applicable in a continuous concept, even at the single vial prototype level. This approach therefore provides an opportunity to provide more insight in the mechanism behind desorption. However, as mentioned before, the mechanistic modeling of desorption has proved to be difficult and failed to include the effect of different freezing regimes on the desorption rate. Another downsize of this approach is of course that these mechanistic models require extensive calibration and validation. Moreover, some parameters such as the heat conduction of the dry layer or enthalpy of vaporization of sorbed water for a certain product are hard to acquire, which limits its possibilities. Using a DOE approach, however empirical, overcomes these difficulties and allows for the construction of a design space for process settings, which would lead to a satisfactory residual moisture content. In the pharmaceutical industry, a low moisture content is often required (<1–2%) to ensure that the shelf life of the product is sufficient. For some products, on the other hand, overdrying can hamper the activity of the active pharmaceutical ingredient or even damage the product. This is especially true for protein-based products where water tends to stabilize their conformational structure. For this purpose, a design space was constructed as an example using the data presented above for a filling volume of 3 mL and a secondary drying processing time of 2 h. The acceptance limit was set to 1% and Monte Carlo simulations were used to include the uncertainty on the factors which were included in the MLR model. The desired residual moisture content was set between 0.8% and 1.2%. The result is a design space for process settings, which would result in an acceptable moisture content with only 1% failure, presented in Figure 6. The advantage of this approach is highly compatible with the concept of continuous freeze-drying since every vial can be subjected to the same desired process settings. This is hardly true for batch freeze-drying since changing the shelf temperature, for example, would not lead to the same change in heat transfer for every vial on the shelf due to the radiation effect from the surroundings [30,31]. For the presented continuous freeze-drying technology, cooling rate and product could be controlled at the single vial level using a PID approach. The small difference between results for the vials, which were processed using the same process settings (center points), shows that this is indeed a valid approach. Additionally, since the constructed MLR model is also able to predict $k_{d}$ in function of the process settings, the process time can be varied and the effect on the final moisture content can be determined using Page’s model. In conclusion, combining inline NIR and a solid experimental design can be used as a valid strategy to optimize secondary drying process settings for the continuous spin freeze-drying technique.

## 4. Conclusions

In this work, a new methodology was presented to study the desorption kinetics during freeze-drying using NIR as an inline PAT tool to measure the change in residual moisture of the sample over time. This approach was used more specifically to study desorption kinetics during the secondary drying phase of an innovative spin freeze-drying technology. The product temperature during secondary drying had the highest positive influence on the desorption rate. Using higher cooling rates during the freezing phase also increased the drying rate while higher filling volumes tended to lower the rate of desorption. The chamber pressure did not affect the desorption process in the range of 10 to 30 Pa. In the future, inline NIR can be used to study desorption kinetics of different formulations and could potentially provide more insight in the mechanism of the desorption process. In addition, the presented approach can be applied to derive the optimal process settings to optimize the desorption rate during the secondary drying step of the continuous freeze-drying process.

## Figures and Tables

**Table 1:** Experimental design and results.

Run	Cooling Rate (°C/min)	Product Temperature (°C)	Filling Volume (mL)	Pressure (Pa)	*k_d_* (1/s^0.5^)	*C_end_* (%)	*C_KF_* (%)
1	9	30	2	10	0.017	1.30	1.19
2	50	30	2	30	0.028	0.59	0.63
3	9	40	2	30	0.026	0.72	0.69
4	50	40	2	10	0.053	0.05	0.22
5	9	30	4	30	0.012	2.20	2.00
6	50	30	4	10	0.021	1.34	1.72
7	9	40	4	10	0.023	0.74	0.85
8	50	40	4	30	0.041	0.17	/
9	29.5	35	3	20	0.023	0.88	0.99
10	29.5	35	3	20	0.024	0.67	0.90
11	29.5	35	3	20	0.021	0.70	0.70

$k_{d}$: kinetic desorption parameter; $C_{end}$: measured residual moisture content (NIR) at the end of the secondary drying process; $C_{KF}$: measured residual moisture content (Karl Fischer) at the end of the secondary drying process; /: missing data.

**Table 2:** Validation parameters of PLS calibration models.

Wavelength Range (nm)	Latent Variables	RMSEcv (%—*w*/*w*)	RMSEP (%—*w*/*w*)	R^2^ (Cummulative)
1100–2200	2	0.254	0.229	0.990
1390–2200	2	0.170	0.220	0.996
1865–2200	2	0.161	0.216	0.996

## Data Availability

All data available are reported in the article.
